# A new corrective technique for Adolescent Idiopathic Scoliosis. Convex manipulation using 6.35mm diameter pure titanium rod followed by concave fixation using 6.35mm diameter titanium alloy

**DOI:** 10.1186/1748-7161-10-S1-O68

**Published:** 2015-01-19

**Authors:** Hidetomi Terai, Hiromitsu Toyoda, Akinobu Suzuki, Sho Douzono, Hiroyuki Yasuda, Koji Tamai, Hiroaki Nakamura

**Affiliations:** 1 Osaka City University Graduate School of Medicine, Japan

## Objective

The purpose of this study was to introduce a new corrective technique for Adolescent Idiopathic Scoliosis (AIS) and its efficacy compared to conventional technique.

## Material & methods

(*Surgical Procedure*) There are two major unique points in our technique; (1) Curve correction is always started from convex side with derotation maneuver and in-situ bending followed by concave rod application. (2) 6.35mm diameter pure titanium rod is used in convex side and the same diameter of titanium alloy rod is used in concave side. Other detailed techniques such as in-situ bending, compression- distraction, derotation maneuver, osteotomies and bone grafting are used in the same manner as previously described.

(*Study methods*) Forty two patients treated surgically from 2008 to 2012 were included in this study and divided into 2 groups; 40 patients (3 men and 37 women, the average age was 15.9) treated with new technique using 6.35mm diameter of different stiffness titanium rods during August 2008 to July 2012 (Group N) and 12 patients (12 women, the average age was 18.8) treated with conventional methods using 5.5mm diameter titanium alloy rods since July 2008 through July 2009 (Group C). All patients had a minimum follow-up of 2 years. Radiographical parameters and peri-operative data were retrospectively collected and analyzed.

## Results

Preoperative main Cobb angles were 56.8° / 60° (Group N/Group C) which improved to 15.2° / 17.1° at the latest follow-up. Correction rates were 73.2% / 71.7% respectively. Thoracic kyphosis increased from 16.8°/16° to 21.3° / 23.4°. There were not significant differences in each parameter. However, mean operating time in Group N (364 min.) was significantly shorter than those of Group C (456 min.).

## Conclusion

The correction rates of new methods in coronal, sagittal and axial plane were same as conventional methods though the operation time was significantly shorter. It had been said that concave manipulation should be started in advance of convex manipulation to avoid worsening of vertebral rotation, however, we proved the efficacy of convex side manipulation in advance of concave side. We considered that it could be possible when only 6.35mm diameter pure titanium rod was used. Our new technique can provide easy and safe surgical correction for AIS.

